# Pediatric resident knowledge, experience, comfort, and perceived competency in providing sibling psychosocial support

**DOI:** 10.5116/ijme.5e63.6a46

**Published:** 2020-03-20

**Authors:** David Buchbinder, Sonam Sidhu, Melissa A. Alderfer, Anne Lown, Russ C. Kolarik, Tommy Wang

**Affiliations:** 1 Department of Pediatrics, University of California at Irvine, Orange, CA, USA; 2 Department of Pediatrics, University of California at Los Angeles, Los Angeles, CA, USA; 3 Department of Pediatrics, Sidney Kimmel Medical College at Thomas Jefferson University, Philadelphia, PA, USA; 4 Department of Social and Behavioral Sciences, University of California, San Francisco, San Francisco, CA, USA; 5 Department of Internal Medicine-Pediatrics, University of South Carolina, Greenville, SC, USA

**Keywords:** Chronic illness, family centered care, brother, sister, resident education

## To the Editor

There is a growing number of children with life-threatening chronic health conditions in the United States.^1^^-^^2^ Care must encompass consideration of psychosocial support for family members.^3^  There is a variety of unmet psychosocial support needs among siblings of children with chronic conditions.^4^^-^^6^ Despite a focus on family-centered care,^7^^-^^8^ it is unclear whether pediatric training programs prepare physicians to address the psychosocial needs of their patients’ siblings.^9^^-^^10^ To address this gap in our knowledge, we sought to determine the extent of pediatric residents’ training, knowledge, experience, comfort, and perceived competence in areas relating to the psychosocial support of siblings. We also aimed to learn from residents their views regarding psychosocial support for siblings, their desire for training, and the content areas in which they are interested.

From June to December 2017, residents were approached for participation during a monthly residency meeting. Completion of the survey was voluntary.  Residents from a single center were asked to provide their year of training if they had been involved in the care of a child whose sibling may have benefited from evaluation and referral for support services, if they had ever evaluated or referred a sibling, and if they had any personal experiences with a sibling of a child with a life-threatening illness.  We asked residents to rate their training, experience, knowledge, competency, and comfort with respect to specific areas of sibling psychosocial care using a Likert-style response scale. Participants indicated their attitudes regarding the psychosocial care of siblings, rating their level of agreement with ten questions created for this study. Finally, participants were asked to rank ten topics (chosen in alignment with published guidelines)^11^ concerning their importance as areas for further training.

Of 90 eligible pediatric residents, 66 completed the survey. This included 22 Post-Graduate Year (PGY)-1 (72% of those approached), 23 PGY-2 (73%), 20 PGY-3 (72%), and 1 PGY-4 (50%). The majority (65%) of residents stated that they had been involved in the care of a child whose sibling may have benefited from evaluation and referral for support services. Only 23% endorsed ever evaluating or referring a sibling. Approximately half (55%), endorsed any personal experiences with a sibling of a child with a life-threatening illness.

Less than half of residents endorsed at least some training, experience, knowledge, competency, and comfort with respect to counseling parents on topics (e.g., emotional, behavioral, quality of life impact on siblings, identifying signs that a sibling has difficulty adjusting) pertinent to the psychosocial care of siblings including ways to talk with siblings about illness, treatment, and family impact as well as talking to siblings when a brother or sister is near death.

A minority (<15%) of residents endorsed at least some training, experience, knowledge, competency, and comfort with respect to counseling siblings on topics (e.g., directly informing and involving siblings, educating donor siblings) pertinent to their psychosocial health including talking directly with siblings about their brother or sister nearing the end of life. Less than one-quarter of residents endorsed at least some training, experience, knowledge, competency, and comfort with screening to identify at-risk siblings as well as providing a referral to psychosocial support services.

The majority (>90%) of residents agreed that primary care providers, specialty care providers, and oncologists have an important role in the provision of psychosocial support for siblings. Across the ten topic areas presented to participants, the top three areas that participants indicated were most important to include in training curricula were (in descending order of importance): 1) counseling parents about siblings’ needs; 2) the types of challenges siblings face when a brother or sister has a serious illness, and 3) screening siblings to determine if they are at risk for problems.

Few pediatric residents endorsed adequate training, experience, knowledge, competence and comfort relating to the counseling of parents and siblings directly on topics pertinent to the psychosocial care of siblings. Moreover, counseling parents and siblings directly was a topic of importance for future training needs. Counseling parents of children with a chronic health condition should include a focus on tending to the siblings’ needs.^12^^-^^13^ For example, pediatric trainees must be able to educate parents to be cognizant of “red flags” that would alert them to a sibling that is having a difficult time adjusting to having a brother or sister with a chronic health condition.  Pediatricians must be able to counsel siblings in ways that facilitate their involvement in the family with greater understanding about the care needs of their ill brother or sister. We must be able to create opportunities to engage siblings through various means such as providing siblings with developmentally appropriate education regarding their brother or sister’s disease and its treatment.^14^   

We found that most pediatric residents lacked training, experience, knowledge, competence and comfort in counseling parents about ways to talk with siblings when their brother or sister was near death as well as communicating with siblings directly when their brother or sister was near death.  The death of a child is one of the most challenging experiences a pediatric resident will face during their training.^15^ Pediatric residents must be able to provide support to soon-to-be bereaved siblings ensuring open and honest communication with these siblings in a developmentally appropriate context.^16^ In the context of bereavement, there is a greater focus on palliative care in pediatrics;^17^ however, residents are still ill-equipped to address the needs of bereaved or soon-to-be bereaved siblings.

Most pediatric resident’s lack training, experience, knowledge, competency, and comfort with respect to conducting a screening assessment to identify at-risk siblings. Moreover, screening siblings to determine if they are at risk for problems was also noted to be a topic of importance for future training. Barriers exist in the context of conducting screening assessments of siblings, including a lack of familiarity with screening tools as well as a lack of time and resources required to conduct assessments.^18^ There is now an increasing number of instruments that may be utilized in the identification of at-risk siblings of children with chronic health conditions.^19^  We must ensure that residents can identify appropriate screening tools as well as utilize, interpret, and apply the results of these screening assessments in their clinical practice.

Although the psychosocial support of siblings has been under the purview of child life specialists and social workers or other healthcare professionals, pediatric residents perceive that they have an essential role to play in the provision of sibling psychosocial support. Perceptions of pediatric residents regarding their role in support of siblings are positive. Despite this, residents face challenges in fulfilling this role, including a lack of training, time, and comfort in the provision of such care. Strategies must be put into place to change our current training system to ensure that these efforts can be supported.

We found that pediatric residents, in general, lacked training, experience, knowledge, competence and comfort in addressing topics that are vital to the provision of sibling psychosocial support.  Findings from this study should inform the development of curricula that will be able to ease the burden of providing psychosocial care to siblings. It is critical that we meet the needs of the siblings’ in order to ensure the delivery of family-centered comprehensive care to patients and their families.

### Conflicts of Interest

The authors declare that they have no conflicts of interest.

## References

[1] Burns KH, Casey PH, Lyle RE, Bird TM, Fussell JJ, Robbins JM (2010). Increasing prevalence of medically complex children in US hospitals.. Pediatrics.

[2] Cohen E, Kuo DZ, Agrawal R, Berry JG, Bhagat SKM, Simon TD, Srivastava R (2011). Children with medical complexity: an emerging population for clinical and research initiatives.. Pediatrics.

[3] Alderfer MA, Kazak AE, Canter K. Families and the healthcare system. In: Fiese B. (editor). APA Handbook of contemporary family psychology. Washington, DC: American Psychological Association; 2018.

[4] McDonald FEJ, Patterson P, White KJ, Butow P, Bell ML (2015). Predictors of unmet needs and psychological distress in adolescent and young adult siblings of people diagnosed with cancer.. Psychooncology.

[5] Patterson P, McDonald FEJ, White KJ, Walczak A, Butow PN (2017). Levels of unmet needs and distress amongst adolescents and young adults (AYAs) impacted by familial cancer.. Psychooncology.

[6] Olivier-D'Avignon M, Dumont S, Valois P, Cohen SR (2017). The needs of siblings of children with a life-threatening illness, part 1: conceptualization and development of a measure.. Palliat Support Care.

[7] Schor EL, American Academy of Pediatrics Task Force on the Family (2003). Family pediatrics: report of the task force on the family.. Pediatrics.

[8] Committee on hospital care and institute for patient- and family-centered care (2012). Patient- and family-centered care and the pediatrician's role.. Pediatrics.

[9] van den Heuvel M, Martimianakis MAT, Levy R, Atkinson A, Ford-Jones E, Shouldice M (2017). Social pediatrics: weaving horizontal and vertical threads through pediatric residency.. BMC Med Educ.

[10] McMillan JA, Land M, Leslie LK (2017). Pediatric residency education and the behavioral and mental health crisis: a call to action.. Pediatrics.

[11] Gerhardt CA, Lehmann V, Long KA, Alderfer MA (2015). Supporting siblings as a standard of care in pediatric oncology.. Pediatr Blood Cancer.

[12] Thompson AL, Young-Saleme TK (2015). Anticipatory guidance and psychoeducation as a standard of care in pediatric oncology.. Pediatr Blood Cancer.

[13] Suris JC, Michaud PA, Viner R (2004). The adolescent with a chronic condition. Part I: developmental issues.. Arch Dis Child.

[14] Barrera M, Atenafu EG, Schulte F, Nathan PC, Hancock K, Saleh A (2018). A randomized controlled trial of a group intervention for siblings of children with cancer: changes in symptoms of anxiety in siblings and caregivers.. Psychooncology.

[15] Hollingsworth CE, Wesley C, Huckridge J, Finn GM, Griksaitis MJ (2018). Impact of child death on paediatric trainees.. Arch Dis Child.

[16] Lockwood B, Humphrey L (2018). Supporting children and families at a child's end of life: pediatric palliative care pearls of anticipatory guidance for families.. Child Adolesc Psychiatr Clin N Am.

[17] Ross MK, Doshi A, Carrasca L, Pian P, Auger JA, Baker A, Proudfoot JA, Pian MS (2017). Interactive palliative and end-of-life care modules for pediatric residents.. Int J Pediatr.

[18] Long KA, Pariseau EM, Muriel AC, Chu A, Kazak AE, Leon M, Alderfer MA (2017). Psychosocial screening for siblings of children with cancer: barriers and preferences.. Clinical Practice in Pediatric Psychology.

[19] Long KA, Pariseau EM, Muriel AC, Chu A, Kazak AE, Alderfer MA (2018). Development of a psychosocial risk screener for siblings of children with cancer: incorporating the perspectives of parents.. J Pediatr Psychol.

